# The Homicide Offender Motive Scale (HOMS): A classification system for homicide motives based on a qualitative systematic review

**DOI:** 10.1111/1556-4029.70170

**Published:** 2025-08-31

**Authors:** John‐Christopher A. Finley, Nisha Sen‐Gupta, Robert E. Hanlon

**Affiliations:** ^1^ Department of Psychiatry and Behavioral Sciences Northwestern University Feinberg School of Medicine Chicago Illinois USA; ^2^ Department of Psychology University of Chicago Chicago Illinois USA; ^3^ Department of Neurology Northwestern University Feinberg School of Medicine Chicago Illinois USA; ^4^ Neuropsychological Associates of Chicago Chicago Illinois USA

**Keywords:** criminal behavior, forensic assessment, homicide, motive, qualitative, systematic review, typology, violence

## Abstract

Many types of homicide motives have been described in the scientific literature. However, inconsistencies regarding how these motives are defined and classified may hinder the ability to understand the driving factors behind homicide. Developing a classification system that defines and organizes commonly used motives into superordinate categories may improve research focused on homicide. The current study sought to develop such a classification system, titled the Homicide Offender Motive Scale (HOMS), based on a qualitative systematic review. Databases including Medline, PsychINFO, and Google Scholar were reviewed to identify all homicide motive studies published prior to February 2024. Multiple reviewers independently assessed the quality of the studies using an empirical appraisal checklist. Seventy‐seven studies comprising 143 motives with varying definitions were included. The reviewers then conducted a thematic analysis to identify descriptive and analytical themes for the 143 motives described in the studies. Following empirical guidelines, the motives were synthesized into 21 descriptive themes with operational definitions and criteria. Perfect interrater reliability of these definitions and criteria was then established in an independent sample of 200 adult homicide offenders. Finally, the 21 descriptive themes were grouped into four distinct analytical themes, which were also partly based on an existing criminological classification system. The 21 descriptive themes represent commonly observed motives across different homicide cases. The four analytical themes are superordinate categorizations of the specific motives. The empirical nature of the HOMS may offer a unified typology of homicide motive for researchers to expand upon.

**Highlights**

- The Homicide Offender Motive Scale (HOMS) is a homicide motive classification system.
- The system composes four distinct superordinate categories that include 21 specific motives.
- The HOMS was based on a systematic literature review of commonly described motives.
- The HOMS had excellent interrater reliability when used in a sample of 200 homicide offenders.
- The HOMS may serve as a unified typology of homicide motive for forensic research.

## 1 INTRODUCTION

In criminal law, ‘motive’ or the ‘general need’ to kill carries significant implications that affect not only the defendant, but the victim's loved ones and society [1]. Consideration of motive during homicide proceedings has shown to influence legal outcomes regarding mitigation, sentencing, and culpability [2]. Expert witnesses, defense attorneys, and prosecutors may rely on interviews or extrapolate from intrapersonal (e.g., psychiatric history) and criminological information (e.g., relationship to the victim) to discern the type and role of motive in homicide. Yet, there is no agreed upon assessment or classification system for homicide motive to be used in criminal law [2, 3]. Although it may never be possible to classify or determine motive with absolute certainty, it is important to develop empirically meaningful classification systems that garner agreement across forensic‐legal professionals. Such classification systems may help expert witnesses, attorneys, and the court better understand the role of motive in homicide cases, to the extent that is possible given the inherent uncertainties in discerning human intentions.

Before any motive classification system is used for the courtroom, it must prove reliable, useful, and gain acceptance within the scientific literature. Such criteria are included in many legal standards for admissibility, such as those reflected by the Daubert standard [4]. In the current study, we propose an empirically informed classification system that researchers can use and expand upon to better evaluate homicide motive and relevant criminological outcomes. Our system is focused on the most commonly described motives within the literature. Developing a classification system for common homicide motives may not only help with ongoing criminological research, but also eventually improve the assessment of motive during homicide proceedings. It is important to emphasize that our classification system should not be used in its current form to assess motive for the courtroom. Much more research is needed before this application of the HOMS is considered. To provide further context for why and how we developed the current motive classification system, we describe existing classification systems and gaps within the literature in the subsequent paragraphs.

Many types of homicide motives have been described in the literature, but several methodological limitations complicate our understanding of how these motives are used and classified. First, few studies offer standardized and empirical criteria for assessing different types of motives [3, 5, 6]. Second, the definition and use of motive vary across professions. For example, research used for policing practices may warrant objective and modifiable motives, whereas psychological research for academic use may identify abstract, theoretical motives [1, 7]. Third, the majority of extant studies rely on small and homogeneous samples of offenders to discern motive. For example, several different motive classifications have been proposed, but exclusively for offenders who commit specific types of homicides, such as filicide [8, 9], parricide [10], intimate partner homicide [11], or sexual homicide [12]. These limitations have resulted in numerous types of motives described in the literature with little consistency in how they are defined and assessed. It is also unclear which of these motives are distinct from each other, posing the concern that researchers are assessing the same motives but with different terminologies. Inconsistent terminology in this context may hinder the translation of research findings to other scientists.

One way to address these issues within the literature is to develop a unified classification system that defines and organizes various motives into discrete yet inclusive superordinate categories. Of the existing classification systems, clustering motives into ‘instrumental’ or ‘expressive’ categories is perhaps the most common approach. Instrumental motive is defined as the need to kill for external gain (e.g., money or sex), whereas expressive motive is defined as an emotional desire to kill [5]. For example, drug‐ or gang‐related motives typically fall within the instrumental category, whereas personal dispute motives are often classified as expressive. This classification system, initially focused on aggressive behavior rather than homicide [13], has proved reliable in criminological research. That is, several important intrapersonal (e.g., psychopathy, psychosis) and criminological factors (e.g., weapon type, victim‐offender relationships) have been consistently associated with the superordinate instrumental‐expressive categories [14, 15, 16, 17, 18, 19, 20, 21]. However, this classification system has also been criticized because the two categories may be overly broad and constrain the investigation of homicide motive [22].

As such, variations of the instrumental‐expressive classification system have been proposed. Some have reconceptualized these categories to describe motive in terms of macro‐ versus micro‐levels [23], and others have included subcategories within the instrumental‐expressive categories [24]. Some have shifted from the instrumental and expressive system altogether, opting instead to categorize motives based on group versus individual activities [25]. Most researchers, however, have developed their own classification systems that are specific to their study sample and aims. These studies often lack an empirical basis for clustering the motives gleaned from their sample into superordinate categories.

Although no perfect motive classification system will ever exist, researchers may better understand and evaluate the role of motive in homicide cases by using a system that organizes common types of motives into empirically meaningful categories. Nevertheless, the vast number of motives described throughout the literature makes it challenging to synthesize them into restrictive categories. Such categories must not be so broad that they provide nonspecific information nor so narrow that they miss overarching patterns regarding motive. There are some existing classification systems that aim to balance the breadth and depth of various criminological factors and may serve as a useful reference for a motive classification system. The Crime Classification Manual (CCM [26]) is one such system that synthesizes numerous, commonly described violent crimes into three superordinate categories: personal cause, sexual homicide, and criminal enterprise. These three categories are more closely associated with offender characteristics and criminal behaviors than the specific crimes, offering a structured approach to understanding violent behavior [26]. Using a similar approach may be helpful for homicide motive classification.

By clustering motives into three or four major categories, researchers could assess the specific types and superordinate themes of motive in an organized and coherent manner. Including three or four major categories may also help overcome issues regarding the simplicity of systems that dichotomize motive into binary constructs (e.g., instrumental versus expressive [22]). Furthermore, developing a motive classification system similar to other criminal classification systems like the CCM could enhance its practical utility. Many criminal classification systems like the CCM focus on crime characteristics (e.g., crime scene staging, offender‐victim relationships, weapon use) in relation to offender characteristics (e.g., personality, demographics, routine activities [27, 28, 29]). Thus, employing a motive classification system that also draws from the characteristics of the crime and offender may provide a practical way to assess motive using data that are already obtained during homicide investigations. Said differently, developing a motive classification system with a similar structure as a classification system like the CCM – both in terms of the number of superordinate categories and type of information used to define the categories – could offer a practical approach for establishing and understanding homicide motive. Although the underlying structure of a motive classification system should have a practical basis for how it is used, the actual motive categories should be empirically derived. One way to achieve this is to use a validated method (e.g., qualitative systematic review) to identify themes across motives that are commonly described in research. An additional benefit of developing a classification system based on commonly cited motives is that it facilitates the use of the system across different types of homicides and research contexts.

Taken together, there are several ways in which researchers can address the aforementioned limitations (i.e., use of overly specific or broad, inconsistently defined, and potentially indiscrete classification motive categories) to improve the assessment of homicide motive. Addressing these limitations can also improve upon prior classification systems and advance the field of forensic research in several ways. First, developing a classification system based on a review of commonly cited motives in the literature could improve the scientific rigor of motive assessment and increase the generalizability of relevant research findings. Second, creating a system with empirically derived definitions of common motives may not only address the lack of consistency in existing motive definitions but also improve the replicability and cross‐disciplinary comparability of research findings. Third, organizing multiple specific motives into three or four superordinate categories could address concerns about systems being too narrow or broad. A two‐tiered system of this kind can also help researchers know which motives correspond with each superordinate category, providing a more intuitive and practical assessment framework. Fourth, this two‐tiered system could help address issues regarding redundancy by grouping overlapping motives into a single superordinate construct, while classifying conceptually distinct motives into discrete constructs. This structure may not only reduce overlap across motive types but also accommodate cases with multiple motives. A system with discrete superordinate constructs could ameliorate some of the statistical modeling issues in the literature (e.g., multiple statistical comparisons within a small sample size) by offering researchers a more parsimonious model of motive. Fifth, developing a classification system with a structure similar to existing systems like the CCM could offer a practical approach to establishing homicide motive since it would be based on data already collected during investigations. These five areas of improvement may help researchers develop a uniform typology of motive that is reliable, practical, and accepted within the scientific community. Such progress could ultimately provide a more effective framework for motive assessment for the courtroom.

The purpose of the current study was to develop an empirically meaningful classification system of homicide motive that addresses these five issues, expands upon existing systems, and ultimately improves the investigation of homicide in research settings. Specifically, we conducted a qualitative systematic review to (1) identify and define commonly used motives, and (2) synthesize the identified motives into three to four distinct superordinate categories that were also partly based on the CCM. By creating discrete superordinate categories that comprise similar groups of motives, we aimed to capture homicide cases associated with multiple redundant motives using just one superordinate category. To evaluate the effectiveness of the classification system in capturing multiple motives and its interrater reliability, we then applied the motive classifications to a sample of adult homicide offenders.

## 2 METHOD

### 2.1 Search procedure

We conducted a qualitative systematic review following the guidelines established by the Preferred Reporting Items for Systematic Reviews and Meta‐Analyses (PRISMA) framework [30]. See Table S1 for details. We searched for peer‐reviewed articles within PsycINFO, Google Scholar, and Medline databases. Reference sections of relevant studies were checked for additional sources. Forward searches of relevant studies were also conducted in Google Scholar. We searched for studies using specific wildcard terms in their title, abstract, or main text, applying Boolean operators to refine the search. The search terms included: “homicid* AND motiv*,” “violent AND motiv*,” “murder AND motiv*,” and “violent intent*.” These terms, combined with the Boolean operator “AND,” were used both individually and in conjunction to systematically identify literature related to homicide motives. We searched for all studies prior to February 2024. This study was not pre‐registered.

### 2.2 Study selection and eligibility criteria

Inclusion criteria were established prior to conducting the literature search process. Studies were included if they (a) investigated any type of homicide motive in an adult (ages ≥18) offender sample, (b) were written in (or translated to) English language, and (c) were published in peer‐reviewed journals (see Table S2 for more details). We first excluded commentaries, conference abstracts, prepublication studies, letters to the editor, and gray literature. We then removed duplicate records. Thereafter, two authors (JF and NSG) independently screened the potentially relevant articles' titles and abstracts against the inclusion criteria. The two authors then independently reviewed the full texts of the remaining articles. Any disagreements were resolved through discussion until a consensus was achieved.

### 2.3 Data extraction and quality assessment

A data collection spreadsheet was used to record the following information: (1) search strategies; (2) inclusion criteria; (3) study characteristics (authors, publication year, article type); (4) sample characteristics (overall sample size, sample sizes for experimental and control groups); and (5) description of the homicide(s), motive(s), and main findings. Two reviewers (JF and NSG) independently extracted this information, compared their findings, and addressed any discrepancies. There were no instances where the reviewers needed to contact the study authors to gather missing information.

We examined the quality of the studies using the Critical Appraisal Skills Program (CASP [31]). Studies were rated individually by two authors (JF and NSG), with CASP ratings ranging from 0 to 10 and higher scores indicating better quality (see Table S3 for more details regarding the quality of studies reviewed). There were few contrasting ratings between raters and each were resolved with discussion. Because we included studies based on a qualitative description of motive(s) rather than qualitative analyses, we modified two items on the CASP. For the CASP item two (“Is a qualitative methodology appropriate?”), we focused on the methodology for defining the motive(s). For item eight (“Was the data analysis sufficiently rigorous?”), we focused on analyses exclusive to the motive as some studies were not solely focused on motive.

### 2.4 Identified studies

**FIGURE 1:**
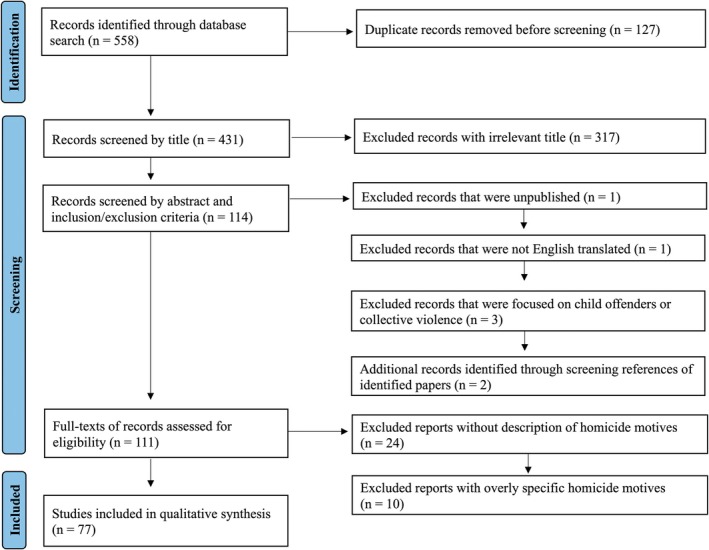
PRISMA flow diagram summarizing the selection process.

As shown in Figure 1, these search procedures returned 558 records, including 127 duplicates. Of the 431 unique records, 317 were removed because their title indicated the paper was entirely unrelated to homicide or homicide motive. Two were removed because they were unpublished (e.g., conference abstracts, notes, unpublished theses/dissertations, editorial letters) or were not English‐translated. Another three were removed because their abstract indicated that the study focused on collective violence or child offenders. However, during this search process, another two studies were identified via existing article references, and were included in the full‐text review. When removing the five studies and adding the two studies, a total of 11 full texts were reviewed. In reviewing the full texts, 24 were excluded because they never actually described a type of homicide motive, and 11 were excluded because they assessed overly specific motives that were clearly unrelated to the majority of the other motives (e.g., mercy killing, family honor). This left 77 studies that met our inclusion criteria.

### 2.5 Thematic analysis

From each of these 77 studies, we extracted the motive definition(s), type of homicide(s) associated with the motive, and any qualitative outcomes associated with the motive to be used in the thematic analysis. We extracted a total of 143 descriptive motives along with the associated homicide and criminological outcomes. We entered the verbatim definitions, homicide type, and outcome associated with the motives into our database, and then conducted a thematic analysis to synthesize themes across them. Following empirical guidelines [32], the analysis involved three stages: (1) line‐by‐line coding; (2) development of descriptive themes; and (3) generation of analytical themes. In the first step, two reviewers (JF and NSG) independently coded each line of the sentences describing the motive and associated information according to their meaning and content. The codes varied in length, ranging from a few words to several lines of text. In the second step, the two reviewers compared and organized the codes into broader categories, referred to as ‘descriptive themes.’ These descriptive themes were essentially a summary of the motive definitions, homicides, and associated outcomes with minimal inferred interpretation. We identified 21 descriptive themes, which were intended to serve as specific motives. The final stage involved the interpretation of these descriptive themes to develop ‘analytical themes,’ which were intended to serve as the superordinate motive categories. In order to create inclusive and distinct analytic themes, we ‘went beyond’ the content of the primary studies, considering information beyond the 21 motive definitions (for details, see [32]). This step demanded more judgment and was harder to execute than the other steps because many motive definitions were similar. We relied on the CCM to serve as a practical framework here, considering how our themes could parallel and be used in conjunction with the CCM. All three author reviewers (JF, NSG, and RH) conducted this stage of the analysis independently and then together. Through these discussions, more abstract, analytical themes emerged. We repeated this process until we were able to abstract enough information that could be defined operationally and included our initial descriptive themes. This final process resulted in four analytical themes. See Table S3 for additional information regarding the motives that were used in this thematic analysis.

### 2.6 Reliability of the descriptive themes in a sample of homicide offenders

The next step was to assess the interrater reliability of the descriptive themes (using our proposed definitions) within a sample of homicide offenders. We also wanted to assess if one of the four analytic themes could sufficiently capture offenders with multiple motives. We did not determine the reliability of the analytical themes because they encompass the descriptive themes; thus, our ratings could have been influenced by our prior knowledge of the descriptive themes. We did not have enough reviewers who were unexposed to the descriptive themes to rate the analytical themes.

Ratings were based on 200 adult offenders convicted of or charged with the first‐degree murder of 363 different victims, 67 of whom were children and 293 of whom were adults. Specifically, 63% of the sample was charged with or convicted of the murder of one individual (*n* = 125), 35% was charged with or convicted of killing multiple individuals in a single instance (i.e., double murders, triple murders, or mass murders; *n* = 70), and the remaining 2% of the sample was convicted of serial murders (*n* = 5). The majority of the sample were males (86%), in their mid‐30s (mean age = 34.48; SD = 12.37), and with partial high school education (mean years of education = 10.74; SD = 2.37). Approximately, 58% of the sample identified as non‐Hispanic Black (*n* = 116), 31% identified as non‐Hispanic White (*n* = 62), 10% identified as Hispanic/Latinx (*n* = 19), and 1% identified as another race (*n* = 3). Based on a prior study using data from this sample [33] the offenders demographic makeup aligns with the general population of violent offenders. The majority (*n* = 110; 55%) of the sample had a history of psychiatric diagnosis and/or treatment (predominately involving symptoms from an internalizing psychiatric, personality, neurodevelopmental, psychotic, or substance use disorder) as well as a history of concussion. All of these individuals were referred for a forensic neuropsychological evaluation for post‐conviction mitigation or the determination of fitness to stand trial, criminal responsibility or sanity, capacity to make a knowing and intelligent waiver of Miranda rights, or mitigating factors during the guilt–innocence phase. Evaluations were conducted by the senior author between2000 and 2020. Evaluations included record review (e.g., police reports, crime scene photos, criminal records, medical records, psychiatric records, educational records, vocational records), clinical interview, and a standardized neuropsychological assessment.

To establish the reliability of the themes, two blinded raters (JF and RH) independently assigned one of the 21 descriptive themes to each offender within our sample. The raters had access to detailed information, including crime scene descriptions, neuropsychological evaluations, court transcripts, and statements made by offenders to police investigators or examiners. Rater 1 (RH) rated the descriptive (themes) motives for all defendants, while Rater 2 (JF) rated half of the defendants (stratified by every other participant to reduce bias). In cases where defendants had multiple motives, raters assigned a primary and secondary descriptive (theme) motive. No defendants were deemed to have three or more motives. We then examined how well the four analytical themes accounted for defendants with multiple descriptive (themes) motives, ensuring that the factors were discrete from each other and even participants with multiple motives were confined to one factor.

## 3 RESULTS

### 3.1 Thematic synthesis

From the 77 studies, we identified 143 descriptive motives. These motives were then refined into 21 descriptive themes that were operationally defined and applied to a sample of adult homicide offenders. When applying these definitions, the interrater reliability for primary and secondary motives was perfect and offenders with multiple descriptive (themes) motives were accounted for by just one of the four analytical themes. Approximately, 27% of the offenders in our sample (*n* = 53) had a primary and secondary descriptive (theme) motive. The most common descriptive motives in our sample were altercation/dispute (*n* = 61), robbery (*n* = 42), or revenge (*n* = 26). Elimination (*n* = 37), revenge (*n* = 11), and concealment (*n* = 8) were the most common secondary motives. Further review and refinement of the descriptive themes resulted in four distinct analytical themes: (1) Personal Motives, (2) Cognitive‐Practical (Instrumental) Motives, (3) Sex‐Based Motives, and (4) Psychotic Motives. As stated above, the review and refinement of these descriptive themes was also partly guided by the CCM. For descriptive purposes, we found that 49% of our sample had Personal motives (*n* = 98), 38% had Cognitive‐Practical (Instrumental) motives (*n* = 76), 6% had Sex‐Based motives (*n* = 12), and 7% had Psychotic motives (*n* = 14). These motives were derived in a structure that can allow for researchers to further investigate the utility of the 21 descriptive themes (i.e., specific motives) in addition to the four superordinate categories. Specifically, this two‐tiered structure can allow for the classification system to be cross‐validated in numerous ways. Table 1 presents the relationship between the four analytical themes and the 21 descriptive themes, including their operational definitions. Below, we expand upon each of the superordinate themes and some of the specific motives they are associated with.

**TABLE 1:** Superordinate categories of the Homicide Offender Motive Scale (HOMS) and specific motives within each category.

Specific motives (descriptive themes)	Operational definitions for motives (descriptive themes)	References from systematic review
Cognitive‐practical (instrumental) motives		
Robbery	During a robbery in which the offender attempts to steal items of monetary value (e.g., a phone, wallet, or car) directly from the victim without consent, they strategically kill the victim during the commission of the crime to successfully complete the robbery and/or avoid detection. The robbery does not involve acquiring drugs, a human, or material items that cannot be physically removed from the victim during the crime (e.g., a home)	[37, 38, 39, 40, 41, 42, 43, 44, 45]
Kidnap	When abducting a victim against their will, either for personal or financial reasons, the offender kills one or more secondary victims that are affiliated with the primary victim, including parents, siblings, caregivers, spouses, or other associates. The offender does this because these victims are thought to interfere with their objective	[26]
Drug	Killing exclusively for the procurement of drugs (during drug‐transactions, drug‐role, and drug‐use events), which differs from gang‐ and robbery‐related homicides	[16, 37, 43, 44, 46, 47, 48, 49, 50, 51, 52, 53, 54, 55, 56]
Gang	Killing for gang‐affiliated factors, regarding “the economic, social, or territorial interests of a gang (e.g., dispute over turf or colors, a gang initiation, internal conflict within a gang for power, or rivalry between two or more different gangs)” [44]	[44, 45, 47, 48, 50, 51, 53, 56, 57, 58, 59, 60]
Property and insurance	Killing for insurance proceeds or physical property as a direct benefit from the death of the victim	[1, 40, 61, 62, 63]
Contract (3rd Party)	Killing as part of a transactional business deal in which one party contracts another to kill a third party. The offender must have no prior relationship with the victim	[5, 46, 64]
Concealment	Killing in an effort to cover up a previously committed crime or homicide; this includes as a means to eliminate a witness, prevent a person from discovery of the crime, or redressing a homicide as an accident	[1, 5, 40, 65]
Suicide by police	An offender killing someone as a means to be killed themselves by law enforcement; this is considered suicide by proxy	[26, 66]
Sex‐based motives		
Sexual sadism	Killing based on a pleasure‐oriented approach where the offender inflicted pain or suffering onto the victim as a means of acting out a sexual fantasy; sexual pleasure is either derived from the act of torture itself or the use of sexual torture on the victim before death	[16, 64, 67, 68, 69, 70, 71]
Sexual degradation	Killing related to the sexual humiliation and degradation of the victim	[72]
Personal motives		
Power and control	Killing where there is a power interest involving the offender exerting physical dominance over the victim in order to seek control; this is shown by the expression of assertion over the victim. The offender sought to experience a feeling of empowerment and a sense of control from the killing	[1, 64, 72, 73, 74]
Better life/Defense	Offender who intentionally kills someone in an effort to improve their own quality of life; the victim must be someone whom the offender knows personally and perceives as directly impairing their quality of life, which is different than killing someone as an immediate act of self‐defense	[25, 64]
Desertion/Termination	Homicide involving romantic victim‐offender relationship in which the death of the victim was in an attempt to prevent the victim from romantically abandoning the offender	[75]
Altercation/Dispute	Killing someone in the heat of the moment due to an inability to control emotions during a spontaneously escalating interpersonal dispute. The offender must believe they have been emotionally harmed (via personal insults, affiliation insults, disrespect, degradation, or criminal acts) by the victim and/or have emotionally suffered as a result of the victim's behavior either prior to or during the altercation	[5, 24, 41, 47, 48, 49, 50, 56, 57]
Revenge	Killing someone in retaliation to a prior incident (with a cooling‐off period) between the offender and victim	[16, 38, 40, 56, 57, 70, 75, 76, 77, 78]
Hate (Non‐Racial)	Killing out of resentment toward a person or from prejudice (not involving race or ethnicity) held against the victim	[38, 63, 73]
Elimination	Killing a person who is considered an obstruction to the offender, as a means to get rid of them for any emotion‐driven reason, such as jealousy of competition, fear of not preserving a secret, or losing romantic partner; this does not include concealing a crime	[26, 38, 64]
Thrill	Killing someone to experience euphoria and emotional/physiological satisfaction associated with the adrenaline rush from the act of homicide. This desire typically arises after killing an initial victim for reasons unrelated to thrill‐seeking	[5, 26, 64]
Annihilation	Compulsive urge to kill multiple victims in a circumscribed location, only occurring spontaneously after the initial homicide. The premonitory sensation or urge before the killing spree is not driven by euphoria or adrenaline‐related excitement. The cooling‐off period between victims can vary	[5, 38]
Psychotic motives		
Command hallucination	Killing someone due to a well‐formed demanding or persuading visual and/or auditory hallucination. Symptoms of hallucinations must be present	[79, 80]
Delusional beliefs	Killing someone to appease or satisfy a nonrationalizing or rationalizing delusion. These delusions can manifest in various forms, including paranoia, grandiosity, envy, religious fervor, or other specific types, but do not take the form of a command hallucination. No auditory or visual hallucination should have been present	[63, 79, 80, 81, 82]

#### 3.1.1 Analytical (superordinate) theme 1: Cognitive‐practical (instrumental) motives

Homicides with motives that are predominantly Cognitive‐Practical are characterized by the formulation of a strategic plan and pragmatic execution of the plan. Because many of these motives are also referred to as ‘instrumental’ within the literature [5], we have chosen to use ‘instrumental’ and ‘Cognitive‐Practical’ interchangeably for the scale; but it should be noted that not all of these motives necessarily involve killing for external gain as the term ‘instrumental’ often implies [5]. Predominantly, Cognitive‐Practical motives are practical, albeit violent, methods of achieving a goal that commonly involves acquiring money or another object with monetary or personal value. Typically, with Cognitive‐Practical motives, the offender and victim do not have preexisting or close personal relationships, though this is not a necessary criterion. The Cognitive‐Practical theme includes subthemes (specific motives) related to robberies, kidnapping, drugs, gang involvement, property/insurance procurement, contracts, and attempts to conceal another homicide, as well as killing to be killed by police.

For example, the ‘robbery’ motive occurs when the offender attempts to steal items of monetary value (e.g., a phone, wallet, or car) directly from the victim. The offender strategically kills the victim during the crime to successfully complete the robbery and/or avoid detection. The offender's belief may be that this will decrease the likelihood that they will be identified, decrease the likelihood that the police will be immediately notified of the crime, or interfere with their ability to obtain the money or product of interest. Another example of a predominantly Cognitive‐Practical motive is ‘kidnapping,’ whereby the offender's objective is to kidnap a victim (e.g., infant, child, adult) for personal possession of the victim or to hold the victim in anticipation of a ransom payment. In order to achieve possession of the primary victim (i.e., target of the kidnapping), the offender kills one or more secondary victims that are affiliated with the primary victim, including parents, siblings, caregivers, spouses, or other associates. The offender does this because these victims are thought to interfere with their objective or because the victims are potential witnesses of the kidnapping. A ‘drug’ motive would be considered if the offender's objective is to obtain illicit substances, money associated with drug sales, or drug purchases that are in the possession of the victim. Typically, the victim is a drug dealer or user whom the offender kills to acquire the drugs or the associated money. These three motives (involving robbery, kidnapping, and drugs) are predominantly Cognitive‐Practical because they involve homicides that are initiated to complete crimes that are not primarily driven by personal or sexual desires or psychotic experiences. The difference between these three specific motives lies within the circumstances that were occurring before the decision to kill was formulated. Concealment is likely to be a common secondary motive to these types of motives. Suicide by police is another Cognitive‐Practical motive, but it is quite different from the previously mentioned examples. This motive occurs when the offender kills a victim with the goal of being killed themselves by police. It may not be uncommon in these cases for the offender to remain armed with a weapon in the location of the dead victim, provoking law enforcement to kill them.

#### 3.1.2 Analytical (superordinate) theme 2: Personal motives

The Personal motives theme consists of specific motives that are primarily driven by prior interpersonal relationships, grievances, or perceived threats posed by the victim. These motives typically involve a personal history between the offender and the victim and may be emotionally salient, retaliatory, or identity‐related. However, these motives, especially those involving a wounded ego, perceived betrayal, or other relational threats, are more likely to involve premeditated and strategic violence rather than emotionally impulsive or affectively dysregulated violence. The idea of Personal motives being more premeditative parallels Meloy's [34, 35] distinction between affective and predatory violence. While most of the motives within this theme (e.g., ‘power and control’ or ‘elimination’) likely involve a form of predatory violence, others may be affective in nature (e.g., ‘thrill’ or ‘annihilation’). The current classification system uses the term “Personal” to describe the interpersonal and relational context in which these motives arise; but it may be helpful to also use Meloy's affective‐predatory distinction as an additional qualifier when classifying these types of motives. Further characterizing the Personal motives as either predatory or affective could provide more specificity about the offender's emotional state and behavioral planning, and whether those factors relate to their motive. Integration of Meloy's criteria [34, 35] was not part of the original development of this classification system, but it may be a useful direction for future research. The subthemes (specific motives) comprising the personal theme include motives predominately regarding revenge, elimination, hate, annihilation, thrill, and altercation/dispute.

For example, with a ‘vengeance’ motive, the offender may believe that they or their significant others have been harmed by the victim, or that they have suffered as a result of the victim's behavior in the distant or recent past. This perceived harm or suffering may be derived from a physical, emotional, financial, interpersonal, sexual, marital, familial, educational, or vocational experience that the offender has had with the victim. The offender commonly harbors resentment toward the victim, but may also have similar characteristics as the victim (e.g., race, gender, sexual orientation), and the killing is motivated by revenge and retribution for the perceived harm the victim previously caused them.

For the ‘altercation/dispute’ motive, the offender believes they have been personally and emotionally harmed by the victim and/or have emotionally suffered as a result of the victim's behavior in the past, including the immediate past or at the time of the altercation. The perceived harm and/or suffering experienced by the offender typically involve personal insults, affiliation insults, disrespect of the offender or the offender's associates (e.g., romantic partners, family members, friends), degradation, or criminal acts (e.g., theft of a vehicle, weapon, drug, money) at the time of the altercation.

The remaining motives in this category are similar to those previously mentioned, being driven to kill for predominantly personal reasons. ‘Elimination,’ however, can stem from various personal reasons to kill, such as fears associated with someone preserving a secret or losing a romantic partner, though it does not include killing to conceal a crime. Our findings suggest that ‘elimination’ may be a common secondary motive. ‘Annihilation’ is also somewhat different from the other personal motives, as it occurs in cases where the offender has a compulsive urge to kill more victims following the initial homicide. This may be most commonly observed in mass murder cases.

#### 3.1.3 Analytical (superordinate) theme 3: Sex‐based motives

The Sex‐Based theme involves homicides that are typically driven by violent sexual desires that may include domination, abuse, restraint, and/or infliction of physical and/or emotional pain during a sexual encounter. Subthemes comprising the predominately Sex‐Based motives include ‘sexual sadism’ and ‘degradation.’

These subthemes include acts of sexually degrading or humiliating the victim. The offender may or may not have a preexisting relationship with the victim, but there might be the presence of altercations or disputes between the offender and victim during or following the sexual act. For example, if the offender's objective is to psychologically dominate the victim by inflicting physical pain, inducing fear, or inducing emotional suffering on the victim immediately prior to, during, or after the sexual contact with them, this would constitute as ‘sexual sadism.’

#### 3.1.4 Analytical (superordinate) theme 4: Psychotic motives

A psychotic theme was included to capture motives involving severe mental illness, where the offender kills primarily in response to persecutory delusions (i.e., fixed false beliefs), threat/override symptoms, and/or specific command hallucinations. It is worth noting that although offenders who experience severe mental illness often present with cognitive impairment, the presence of cognitive impairment (including intellectual disability or major neurocognitive disorder) in itself does not mean the offender meets criteria for this motive. By nature of the offender's mental state, they may not be able to coherently relay why they killed the victim after the matter. However, some researchers have identified recurring themes related to these psychotic experiences, such as Satanic/antichrist/demonic‐ or God‐themed delusions that compel the offender to kill [36].

These four superordinate motive themes were designed to capture various homicide motives that are commonly observed across various types of homicide cases. However, in our systematic review, we also identified motives associated with certain types of homicide that were too specific to include in the thematic analysis.

## 4 DISCUSSION

Based on findings from the qualitative systematic review and an existing classification model (i.e., CCM), we propose a classification system for homicide motive, titled the Homicide Offender Motive Scale (HOMS). Our systematic review identified 143 different types of motives with varying definitions. We synthesized these motive types into 21 descriptive themes. We defined and established the reliability of these descriptive themes in a sample of adult homicide offenders. We then clustered these 21 descriptive themes into four analytical themes based on characteristics of the crime and offender, which were partly based on the CCM and similar criminal classification systems [26]. The 21 descriptive themes represent and define commonly observed motives across different homicide cases. The four analytical themes are superordinate categorizations of the specific motives. This classification system may be used in several ways, but here we focus on two general applications for its use in research. First, it may be used to help identify the offender's primary or secondary (or tertiary, etc.) motive based on the definitions of the 21 descriptive themes (Table 1). Second, the superordinate categorization of the motives can be used to provide a broader, thematic classification of the motives.

### 4.1 Study implications

Although the HOMS requires additional development and should not be used in its current form to assess motive for the courtroom, it has the potential to address five key limitations in the literature and among other existing classification systems. First, prior research has commonly relied on theoretical constructs, small and homogeneous samples, or sample‐specific classification systems to assess motive. These methodological issues limit the generalizability and replicability of motive‐related research across different contexts. The HOMS addresses this limitation as its classification structure is grounded in a comprehensive review of 77 empirical studies. By synthesizing commonly reported motives into themes derived from diverse samples and contexts, the HOMS may offer a more robust and generalizable framework.

Second, prior studies often lack clear, reproducible criteria for classifying various motives [5, 6]. Researchers may use different terms for similar motives or apply the same motive label in conceptually distinct ways. This lack of consistency and standardization undermines the reliability of motive‐related research. The HOMS addresses this limitation by providing operational definitions for each of the specific motives. These definitions were not only based on empirical literature but also evaluated in an independent sample of homicide offenders. A classification system with empirically derived operational definitions for common motives could improve the consistency of nomenclature across research, which may help improve the replicability and comparability of motive‐related findings across disciplines.

Third, existing classification systems have been criticized for including overly specific or overly broad motive categories [22]. Narrow systems can limit generalizability, while broad ones can obscure meaningful differences between distinct motive constructs by sometimes forcing researchers to dichotomize motives into binary categories that may lack important nuances. The HOMS helps address this problem by offering a two‐tiered structure: (1) twenty‐one descriptive themes that capture common and specific motives; and (2) four superordinate categories that group conceptually similar specific motives into higher order constructs. This design helps balance the sensitivity and specificity of motive and its use as a predictive variable in criminological research. Furthermore, existing categorical systems often do not provide a clear mapping of how specific motives align with their broader categories. This lack of clarity makes it challenging to understand how specific motives described by some researchers (e.g., revenge, thrill) relate to broader motive constructs (e.g., instrumental, expressive) described by others. The HOMS explicitly links each specific motive to a superordinate category, allowing for transparent and tangible classification. This two‐tiered structure provides researchers with clearer guidance, which may increase the consistency in how motives are classified in research, especially when coding cases with multiple or ambiguous motives.

Fourth, existing classification systems are not systematically designed to accommodate offenders with multiple motives. This is problematic given that offenders may have more than one motive. For example, an offender's primary motive might be related to kidnapping, but they may also have a secondary motive of concealment, such as killing a bystander who incidentally witnesses the kidnapping to hide the initial crime. Without a system that accommodates multiple motives, researchers in this example would have to decide whether to focus on the kidnapping or concealment motive. Focusing on one motive over another could lead to the loss of potentially important information, while analyzing each motive separately would require more statistical analyses that might only complicate findings. The HOMS, which has superordinate categories comprising various yet similar types of motives, was designed to capture multiple motives with a single discrete category (i.e., Cognitive‐Practical, Sex‐Based, Personal, or Psychotic). As such, researchers may be able to assess the broader thematic construct across multiple motives (e.g., Cognitive‐Practical), rather than focusing exclusively on one specific motive over another (e.g., ‘concealment’ versus ‘kidnap’). Grouping similar types of motives into distinct categories in this manner also helps reduce the chance of examining multiple, redundant motives in research, which can render biased findings. Many motives have been described in the literature, but it is unclear which of them are distinct from each other, posing the concern that researchers are assessing the same motives with different terminologies. Using more parsimonious statistical models with fewer motive categories may be especially helpful for studies with small sample sizes (by reducing the number of individual comparisons and increasing statistical power), which is also a common problem in this area of research. Although the HOMS was designed to provide a construct that accounts for offenders with multiple motives, it is still possible that offenders will have multiple motives that span more than one of the categories. For example, an offender with psychosis may harbor the delusion that their romantic partner is unfaithful, leading them to murder their partner in a jealous rage, conceal the body for practical reasons, and even engage in sexually humiliating behavior before, during, or after the crime. Such a scenario would place the offender's motive in all four categories. In other words, the HOMS does not offer a complete solution to this issue.

A fifth limitation in the literature pertains to the practicality of existing motive classification systems. If a classification system relies on data that are difficult to access, it will not be applicable across professions and disciplines. A motive classification system that is based on the characteristics of the crime and offender could allow investigators to assess motive using data that is already obtained during homicide investigations. By improving upon these five issues, researchers may eventually devise a uniform typology of motive that is reliable, practical, and well‐accepted within the scientific literature. The HOMS has the potential to address these issues to some extent, but it is not intended to be an exhaustive system that perfectly resolves all of these issues or replaces existing classification systems. Rather, this version of the HOMS can serve as an initial framework for improving the assessment of common homicide motives.

### 4.2 Limitations and future directions

Although the HOMS offers potential, there are several practical and methodological limitations that must be considered. Thus, it is important to re‐emphasize that this classification system should not be used in criminal‐legal work without additional research. With regard to the systematic review, we cannot rule out the possibility that we missed some relevant studies as we did not search the gray literature or non‐peer‐reviewed articles. There is also public and government‐regulated data regarding homicide that was not included in this study but may be useful in understanding motive typology. Along these lines, highly specific motives, such as mercy killing and family honor, were excluded at the outset of the review to maintain consistency and generalizability across the classification system. However, their omission may limit the comprehensiveness of the HOMS in capturing rare but meaningful subtypes of motive. Future research could expand the HOMS by incorporating more specific motives (e.g., mercy killing and family honor) to determine whether they warrant inclusion as additional descriptive or analytical themes within an expanded typology.

As with any qualitative systematic review, the validity of our thematic synthesis may be biased by our interpretation. The general structure of the classification system was biased given that it was largely based on the CCM; but the thematic synthesis of the superordinate categories and specific motive types was independent of the CCM. Furthermore, we followed extensive guidelines to verify the consistency in our interpretation and data extraction, and also established the reliability of our ratings in a sample of offenders. Finally, it is likely that some of the specific motives overlap to some extent, depending on the context of the homicide. Although the superordinate categories were intended to account for this overlap, it is important to recognize that some of the specific motives may align better with another superordinate category than the one in which they were initially classified Therefore, it is possible that both the specific motives and the superordinate categories do not represent entirely discrete constructs. Another inherent limitation of this qualitative study is that we cannot provide quantifiable evidence to demonstrate that the superordinate constructs offer any improvement over those provided by existing systems. Additional research is needed to determine whether the superordinate categories improve the reliability and validity of motive assessment, above and beyond those within existing classification systems as well as using the specific motives alone.

There are also potential practical limitations of the HOMS that must be clarified. For example, it is unclear if the HOMS can be used alongside information regarding the type of homicide. Consider, for instance, a parent who kills a child due to religious (nonpsychotic) conviction, to avoid caregiving responsibilities, or as retaliatory punishment toward the co‐parent. In these instances, the general type of homicide would be filicide. And while one could argue that the motives in these instances fall under the ‘Personal’ superordinate category (given they reflect themes of ‘elimination,’ ‘power and control,’ or ‘revenge’), this may not apply to all instances of filicide. Given that the type of homicide may provide additional and useful information, future research may focus on using homicide type as an additional qualifying variable for motive classification. That is, once the specific motive (e.g., ‘revenge’) and superordinate category (e.g., ‘Personal’) are identified, then the type of homicide (e.g., filicide) is indicated to provide additional context. The incorporation of general types of homicide warrants further consideration, as this information is critical to each case. We recommend that future studies explicitly examine how filicide, parricide, and neonaticide can be integrated into the HOMS framework, given their relatively frequent occurrence. Similarly, other qualifiers (e.g., affective versus predatory) may be considered to improve the specificity and practical applicability of the HOMS classification. Another practical issue that deserves focus is how to assess homicide offenders with multiple motives spanning two or more superordinate categories. Although definitions are provided for each motive, it would be helpful to configure more precise criteria and modes of assessment needed to identify them and determine which are the primary versus secondary motives. Based on prior research, most assessment strategies rely on defendant or witness testimony to determine motive. However, some research [3] suggests that homicide motive should be assessed with additional sources of data. Such factors should be considered in future research investigating the utility of the HOMS.

Given these limitations, it is important to reiterate that the HOMS is not a perfect solution to existing assessment methods, and the use of the superordinate categories (i.e., Cognitive‐Practical, Sex‐Based, Personal, or Psychotic) may not prove useful over time. We encourage researchers to consider alternative approaches to these categories (e.g., using general homicide type instead of the superordinate categories) and empirically evaluate which approach is most effective (i.e., the superordinate categories or something else). In determining whether the superordinate categories should be maintained, it will be especially important to evaluate how well they are associated with homicide risk and relevant criminological factors as compared with other methods. It will also be crucial to determine if the superordinate categories improve the prediction of relevant criminological outcomes, above and beyond the specific motives.

## 5 CONCLUSIONS

The HOMS is an empirically based classification system that retreads much of the existing literature and is designed to serve as a unified typology of homicide motive. It is not intended to be an exhaustive classification system, but rather as an initial method for improving the assessment of common homicide motives in which future research can expand upon. It is also important to acknowledge that the motive of a homicide offender can almost never be determined with certainty, and the classification system here provides no exception. Constructive criticisms, remarks, suggestions for alternative versions, and cross‐validation studies are welcomed to refine this system.

## CONFLICT OF INTEREST STATEMENT

The authors have no conflicts of interest to declare.

## Supporting information


Table S1.

## Data Availability

Extracted data used for all statistical analyses can be found in the Tables S1–S3.
