# Supplementary material for: The Homicide Offender Motive Scale (HOMS): A classification system for homicide motives based on a qualitative systematic review
Source: J Forensic Sci. 2025 Aug 31;70(6):2129–42. doi: 10.1111/1556-4029.70170 (PMC12584117; doi:10.1111/1556-4029.70170)
Supplement: Supplementary file 1 — Table S1. [file JFO-70-2129-s001.docx]

TABLE S1 PRISMA 2009 checklist.

| **Section/topic** | **#** | **Checklist item** | **Reported on page #** |
| --- | --- | --- | --- |
| **TITLE** | | |  |
| Title | 1 | Identify the report as a systematic review, meta-analysis, or both. | 1 (in Title Page doc) |
| **ABSTRACT** | | |  |
| Structured summary | 2 | Provide a structured summary including, as applicable: background; objectives; data sources; study eligibility criteria, participants, and interventions; study appraisal and synthesis methods; results; limitations; conclusions and implications of key findings; systematic review registration number. | 2 |
| **INTRODUCTION** | | |  |
| Rationale | 3 | Describe the rationale for the review in the context of what is already known. | 3, 4, 5, 6 |
| Objectives | 4 | Provide an explicit statement of questions being addressed with reference to participants, interventions, comparisons, outcomes, and study design (PICOS). | 6 |
| **METHODS** | | |  |
| Protocol and registration | 5 | Indicate if a review protocol exists, if and where it can be accessed (e.g., Web address), and, if available, provide registration information including registration number. | 6 |
| Eligibility criteria | 6 | Specify study characteristics (e.g., PICOS, length of follow-up) and report characteristics (e.g., years considered, language, publication status) used as criteria for eligibility, giving rationale. | 6, 7 |
| Information sources | 7 | Describe all information sources (e.g., databases with dates of coverage, contact with study authors to identify additional studies) in the search and date last searched. | 6 |
| Search | 8 | Present full electronic search strategy for at least one database, including any limits used, such that it could be repeated. | 6, S2 |
| Study selection | 9 | State the process for selecting studies (i.e., screening, eligibility, included in systematic review, and, if applicable, included in the meta-analysis). | 8 |
| Data collection process | 10 | Describe method of data extraction from reports (e.g., piloted forms, independently, in duplicate) and any processes for obtaining and confirming data from investigators. | 7, 8 |
| Data items | 11 | List and define all variables for which data were sought (e.g., PICOS, funding sources) and any assumptions and simplifications made. | 7, 8, 9 |
| Risk of bias in individual studies | 12 | Describe methods used for assessing risk of bias of individual studies (including specification of whether this was done at the study or outcome level), and how this information is to be used in any data synthesis. | NA |
| Summary measures | 13 | State the principal summary measures (e.g., risk ratio, difference in means). | NA |
| Synthesis of results | 14 | Describe the methods of handling data and combining results of studies, if done, including measures of consistency (e.g., I^2^) for each meta-analysis. | NA |

Page 1 of 2

| **Section/topic** | **#** | **Checklist item** | **Reported on page #** |
| --- | --- | --- | --- |
| Risk of bias across studies | 15 | Specify any assessment of risk of bias that may affect the cumulative evidence (e.g., publication bias, selective reporting within studies). | NA |
| Additional analyses | 16 | Describe methods of additional analyses (e.g., sensitivity or subgroup analyses, meta-regression), if done, indicating which were pre-specified. | 8, 9, 10, 11 |
| **RESULTS** | | |  |
| Study selection | 17 | Give numbers of studies screened, assessed for eligibility, and included in the review, with reasons for exclusions at each stage, ideally with a flow diagram. | 7 |
| Study characteristics | 18 | For each study, present characteristics for which data were extracted (e.g., study size, PICOS, follow-up period) and provide the citations. | 8, S2, S3 |
| Risk of bias within studies | 19 | Present data on risk of bias of each study and, if available, any outcome level assessment (see item 12). | NA |
| Results of individual studies | 20 | For all outcomes considered (benefits or harms), present, for each study: (a) simple summary data for each intervention group (b) effect estimates and confidence intervals, ideally with a forest plot. | NA |
| Synthesis of results | 21 | Present results of each meta-analysis done, including confidence intervals and measures of consistency. | NA |
| Risk of bias across studies | 22 | Present results of any assessment of risk of bias across studies (see Item 15). | NA |
| Additional analysis | 23 | Give results of additional analyses, if done (e.g., sensitivity or subgroup analyses, meta-regression [see Item 16]). | 10, 11, 12, 13, 14, 15 |
| **DISCUSSION** | | |  |
| Summary of evidence | 24 | Summarize the main findings including the strength of evidence for each main outcome; consider their relevance to key groups (e.g., healthcare providers, users, and policy makers). | 15, 16 |
| Limitations | 25 | Discuss limitations at study and outcome level (e.g., risk of bias), and at review-level (e.g., incomplete retrieval of identified research, reporting bias). | 18, 19 |
| Conclusions | 26 | Provide a general interpretation of the results in the context of other evidence, and implications for future research. | 18, 19 |
| **FUNDING** | | |  |
| Funding | 27 | Describe sources of funding for the systematic review and other support (e.g., supply of data); role of funders for the systematic review. | NA |

*From:*  Moher D, Liberati A, Tetzlaff J, Altman DG, The PRISMA Group (2009). Preferred Reporting Items for Systematic Reviews and Meta-Analyses: The PRISMA Statement. PLoS Med 6(7): e1000097. doi:10.1371/journal.pmed1000097

| TABLE S2 Inclusion criteria. | |
| --- | --- |
| **Criteria** | **Notes** |
| Population | adult (ages ≥18) homicide offenders |
| Phenomena of Interest | any type of homicide motive |
| Comparators | Not applicable |
| Outcomes | The outcomes will be generated using emergent coding, but are expected to include definitions, criteria, experiences, views, and perceptions of offenders, families, scholars, or forensic-legal practitioners on homicide motive |
| Settings | Any |
| Sub-groups | Not applicable |
| Study Types | Systematic reviews of qualitative studies  Qualitative studies that collect data from focus groups or interviews  Studies that collect qualitative data from questionnaires / surveys / vignettes  Mixed method study designs (including qualitative element) |
| Countries | Any |
| Timepoints | Any before February 2024 |
| Other Exclusions | Non-English translated reports |

| TABLE S3 Details of studies included in the thematic analysis. | | | |  |
| --- | --- | --- | --- | --- |
| **Author, Year** | **Motive Type** | **Homicide Type** | **Sample Size** | **CASP Rating** |
| Aarten & Liem, 2023 | Intimate partner, domestic, non-criminal disputes, organized crime and drug trade, robbery, mental illness or sexual, unknown | Mixed | *n* = 5170 victims | 9 |
| Antar, 2023 | Delusions, revenge, jealousy, wife leaving, economic problems, previous conflicts, terrorism, family honor | Mixed | *n* = 128 offenders (72 were deemed not guilty by reason of insanity and 56 were deemed guilty; data obtained from homicide cases in Israel) | 10 |
| Aziani & Persurich, 2023 | Criminal-instrumental, intimate-expressive, unknown | Mixed | *n* = 458 victims | 8 |
| Bahary & Léveillée, 2022 | Quarrel/dispute, criminality, mental illness, sexual | Mixed | *n* = 76 cases | 10 |
| Beauregard et al., 2007 | Anger, sadism | Sexual homicide | N/A (commentary/review) | 9 |
| Beech et al., 2005 | Carry out fantasies, grievance, avoid detection | Sexual homicides | *n* = 28 victims (homicides that resulted in serving mandatory life sentences in the United Kingdom)  *n* = 28 offenders | 10 |
| Bell & McBride, 2010 | Anger/revenge, domestic/romantic, direct interpersonal conflict | Mass murder-suicides | N/A (commentary/review) | 10 |
| Bijleveld & Smit, 2006 | Criminal contract, drug-related, criminal other, sexual, robbery, disputes inmates, disputes acquaintances, disputes strangers, psychotic, unknown | Mixed | *n* = 295 cases (homicide incidents that occurred in the Netherlands in the year 1998; 246 with victim-offender relations data and 49 missing offender data; final descriptive results included 202 homicide incidents)  *n* = 230 offenders  *n* = 225 victims | 9 |
| Bryant & Cussen, 2015 | Revenge, jealousy, desertation / termination, domestic argument, money, drugs, alcohol-related argument, other argument, sexual vilification, sexual gratification, no apparent motive, unknown | Mixed | *n* = 511 victims  *n* = 532 offenders | 8 |
| Chesler, 2010 | Honor | Mixed | *n* = 172 cases (honor killings obtained via global English-language media across 29 countries and territories)  *n* = 230 victims (130 in Muslim world and 100 in the Western world (67 in Europe and 33 in North America)) | 10 |
| Cook, 1987 | Robbery-related | Mixed | *n* = 2,086 victims (based on FBI’s Uniform Crime Reports Statistics for 1981, which comprised 20,053 homicide cases, 2,086 of which were robbery-related homicides) | 10 |
| Coyne-Beasley et al., 1999 | Drug-related, altercations, retaliations, reckless behavior, bystander, robbery, broken relationship, gang-related | Mixed | *n* = 419 victims (from homicides listed in the North Carolina Medical Examiner database from 1990 to 1995 ranging in age from 11 to 18) | 9 |
| Crabbé et al., 2008 | Concealment, greed, domestic argument, revenge, family honor, jealousy, sexual, power reassurance, power assertive or entitlement, anger / retaliation, sadistic or anger excitation, profit or material gain, gang, drug, felony, argument, familial / domestic, criminal enterprise, personal cause, group cause, contract, robbery, inmate disputes, acquaintance disputes, psychotic, visionary, mission oriented, hedonistic, thrill, comfort, lust, power control, love, terror | Mixed | N/A (commentary/review) | 10 |
| Cunningham et al., 2010 | Gang, racial animosity, revenge, altercation/argument, mental illness, intoxication, sexual conflict, victim was a child molester, victim was an informant / drug dealer | Inmate-related homicide | *n* = 35 victims  *n* = 52 offenders | 10 |
| Decker & Curry, 2002 | Gang-related | Mixed | *n* = unknown (via case files from St. Louis Metropolitan Police Department Homicide Division and Gang Intelligence Unit originating in 1989) | 10 |
| Decker, 1996 | Money gain, personal cause | Mixed homicides based on relationship with victim and perpetrator | *n* = 792 cases (homicides recorded by the St. Louis Metropolitan Police Department between 1985 and 1989) | 9 |
| Douglas et al., 2013 | Criminal enterprise, personal cause, sexual, group cause | Mixed | N/A (crime classification manual) | 9 |
| Felthous, 2008 | Non-rationalizing delusion | Mixed | N/A (commentary/review) | 8 |
| Frei & Ilic, 2020 | Revenge, loyalty, psychosis, felony, material gain, elimination, annihilation, hate | Mass murders | *n* = 35 cases (mass murders (3 or more victims in one event) that occurred in Switzerland between 1972 and 2015; 20 familicides, 9 public mass murders, 6 felony-associated mass murders)  *n* = 35 offenders (19 familicide perpetrators were male and 1 familicide perpetrator was female ranging in age from 19 to 57, 9 public mass murders, 6 felony-associated mass murders)  *n* = 145 victims (82 by familicide, 44 by public mass murder, 19 by felony-associated mass murder) | 9 |
| Getoš Kalac et al., 2022 | Clear, unclear (which comprises greed, jealousy, revenge, hate, defense, vigilantism, lack of care, on request) | Mixed | *n* = 2073 cases (data obtained from the Balkan Homicide Study dataset) | 10 |
| Green, 2023 | For profit, to further criminal endeavor, to effect public policy or legal processes, animosity towards victim's characteristics, to assert cruel power over another | Aggravated homicides | N/A (commentary/review) | 9 |
| Grubin, 1994 | Anger | Sexual homicide | *n* = 103 cases (convicted sexual homicides and rapes committed against women; across 6 English prisons)  *n* = 142 offenders (21 men convicted of sexual homicide, 90 men convicted of a single rape, 26 men convicted of multiple rapes, and 5 men convicted of rape that had a second victim) | 9 |
| Gruenewald et al., 2013 | Domestic, drug-related, gang-related, dispute, robbery, other | Mixed | *n* = 866 victims (homicides occurring in Newark, New Jersey between 1997 and 2007 that were covered in the leading local print news “The Star-Ledger”) | 9 |
| Gurian, 2011 | Pleasure-oriented, purpose-oriented | Female-perpetrated serial | *n* = 65 cases (homicides that occurred between 1900 and 2006 across the globe involving female serial homicide offenders of which 30 were committed with a partner and 35 were committed alone; locations include Australia, Austria, Belgium, Canada, France, Greece, India, Mexico, Russia, Spain, United Kingdom, and United States of America)  *n* = 134 offenders (99 partnered offenders (44 females with 55 males) and 35 solo female serial homicide offenders) | 9 |
| Hachtel et al., 2021 | Vengeance, argument, financial gain, sexual, sadistic, filicide | Mixed | *n* = 569 cases (homicides identified by Law Enforcement Assistance Package database in Victoria, Australia that occurred between 1997 and 2005; 64 unsolved)  *n* = 435 offenders (196 convicted of murder, 159 convicted of manslaughter, 29 murder-suicides, 26 found not guilty due to mental impairment, 25 convicted of filicide; 380 male (276 committed alone and 104 with co-offender) and 55 female (40 committed alone, 11 co-offender was male, 4 co-offender was female too); 43 with diagnosed psychosis) | 10 |
| Herzog, 2004 | Commission of a crime, political dispute, family honor, financial dispute, romantic infidelity, domestic abuse, euthanasia, saving money, high-speed driving, error in medical diagnosis, error in discretion, prevention of danger, prevention of escape | First-degree murder, manslaughter, negligent homicide, justified homicide | *n* = 805 adults (sample did not include offenders or victims, but rather a random population of Israeli adults were asked about 10 random hypothetical homicide scenarios involving motive) | 9 |
| Holmes & Holmes, 2009 | Visionary, demand-hallucinations, mission-oriented, elimination, hedonistic, thrill seeking, violence pleasure, comfort, better life, lust, sexual arousal / gratification, power-control, control | Serial homicide | N/A (commentary/review) | 9 |
| Joyal et al., 2004 | Specific delusions | Mixed | *n* = 58 convicted men with schizophrenia or schizoaffective disorder | 10 |
| Keppel & Walter, 1999 | Power assertive, increasing aggression with the victim ensures control and power, power reassurance, acts out fantasy and seeks reassurance from the victim, anger-retaliation, seeks revenge for his anger towards another person by attacking a symbolic person, anger-excitation, engages in prolonged torture, exploitation, and/or mutilation, which energizes the killer's fantasy | Serial sexual homicide | *n* = 2,476 cases (sexually related murders where the offender was serving time in prison; data collected in October 1995 via the Michigan Department of Corrections; 904 power-assertive, 807 anger-retaliatory, 599 power-reassurance, 166 anger -excitation)  *n* = 2,476 offenders (currently serving prison time for sexual homicides; at point of data collection in October 1995 there were 5,928 prisoner serving sentences for homicides; total prison population in Michigan Department of Corrections was 41,584) | 9 |
| Kerr et al., 2013 | Anger, sadism, deviant sexual fantasy, power assertive | Sexual homicides | N/A (commentary/review) | 10 |
| Kingree & Tanner, 1994 | Obtaining life insurance (financial gain) | Mixed | N/A (commentary/review) | 8 |
| Koç, 2022 | Micro-level, macro-level, dispute, domestic violence, attempted divorce, refusal to reconcile, divorce, suspicion of cheating, jealousy, material reasons, demand for separation, custom/honor, other | Femicide | *n* = 1,000 cases | 10 |
| Kristoffersen et al., 2014 | Jealousy, anger of female ending relationship, stop abuse, drinking arguments, psychiatric disorder | Mixed | *n* = 196 victims | 10 |
| Kubrin & Ousey, 2009 | Altercation, felony, drug, gang | Mixed | *n* = 206 cities (homicide data collected across the United States for cities with a minimum population of 100,000 people in 2000 via Federal Bureau of Investigation Uniform Crime Reporting program between 2000 and 2002) | 8 |
| Kubrin, 2003 | Heat of anger, domestic violence, robbery, drug-related, child abuse, rape/sexual, hate, psychotic, retaliation, self-defense, accident | Mixed | *n* = 1,557 cases (1,045 general altercation, 273 felony, 142 domestic male-on-female, 97 domestic female-on-male homicides using tract-level census data from St. Louis in 1990) | 8 |
| Kuhns & Maguire, 2012 | Domestic, robbery, other (the other motive primarily comprised offenders with “street” motives) | Mixed | *n* = 661 victims | 9 |
| Langevin et al., 1988 | Sexual release, anger, both sex and anger, fear, other, unknown | Sexual and nonsexual homicides (and sexual assault) | *n* = 39 offenders (all male; 13 sex killers, 13 non-sex killers, 13 nonhomicidal sexual aggressors)  *n* = 199 victims (99 for sex killers in which 92% were female and 69 are the sole victim; 100 for non-sex killers in which 38% were female and 92 are the sole victim) | 9 |
| Levin & Fox, 1996 | Revenge, love, profit, terror | Mass murder | *n* = ~1,500 victims (data were based on the Supplementary Homicide reports of the FBI for the years 1976-1989)  *n* = >400 offenders | 9 |
| Levin & Fox, 2017 | Power-control-sex, revenge-hate, loyalty, profit, terror-infamy | Serial | N/A (commentary/review) | 10 |
| Liem et al., 2009 | Killing as a result of physical abuse, altruism, means of reprisal again a(n) (estranged) partner for filicides, fear of abandonment, or narcissistic rage for uxoricides, mental illness, domestic problems, relationship problems for parasuicides | Extrafamilial and intrafamilial homicide-parasuicides | *n* = 682 cases (80 homicide-parasuicides, 441 homicides, 161 parasuicides in the Netherlands between 1992 and 2001; domestic-related)  *n* = 668 offenders (77 homicide-parasuicide, 430 homicide-only, 161 parasuicide-only perpetrators) | 8 |
| Liem, 2010 | Anger, paranoia, fear of detection, exposure | Homicide-suicides | N/A (commentary/review) | 7 |
| Mafullul et al., 2001 | Group activity, individual activity | Premeditated and non-premeditated homicides | *n* = 118 offenders (homicide offenders who were convicted ranging in age from 14 to 65 and who were located in the Jos federal prison in Nigeria) | 9 |
| Maguire et al., 2010 | Domestic, robbery, drug / gang, revenge, altercation | Mixed | *n* = 242 interviews (within the Trinidad and Tobago Police Service along with historical data between 1988 and 2005 from the Homicide Bureau of Investigations)  *n* = 113 interviewees (62 employees, 23 from other criminal justice agencies, 13 from other government agencies, 8 community activists and faith community members, 5 gang leaders, 2 gang members) | 10 |
| Mares, 2010 | Gang-related, intimate, robbery-related, drug-related, non-gang-related | Gang-related homicides, intimate killings, and robbery-related homicides | *n* = 8,809 cases (7,506 nongang-related and 1,303 gang-related investigated by the Chicago Police Department between 1985 and 1995; non-gang includes 1,020 drug-related, 969 robbery-related, and 759 intimate-related homicides) | 8 |
| Martinez et al., 2003 | Drug-related, intimate, escalation, robbery, other felony, unknown, other | Mixed | *n* = 1,847 victims (homicides in Miami between 1980 and 1990)  *n* = 1,566 offenders | 9 |
| Marwah (2014) | Gain, property dispute, personal vendetta or enmity, love affairs/sexual causes, dowry, lunacy, witchcraft, terrorism/extreme violence, political reasons, communalism, caste conflict, class conflict, and other | Mixed | *n* = 33,335 cases (findings were based on data from 2000-2010 from The National Crime Records Bureau [2013] dataset^2^) | 9 |
| Matejkowski et al., 2008 | Hatred, retaliation, animosity, revenge, gain of money/property, rage / anger, gratification, relation to other crimes, delusional, suicidal | Mixed | *n* = 130 victims (homicides occurred in Indiana, United States between 1990-2002)  *n* = 95 suspects with severe mental illness | 9 |
| McKee & Egan, 2013 | Psychosis, retaliation / revenge, rejection, mercy killing, accidental | Maternal filicides / neonaticides of biological children (ages <16) | *n* = 21 offenders (women in the United Kingdom convicted of maternal filicide (mothers who killed their biological children) ranging in age from 14 to 47)  *n* = 24 victims (13 sons and 11 daughters ranging in age from newborn to 7) | 9 |
| Meloy et al., 2013 | Vengeance, result of humiliation, gain (notoriety) | Mixed | *n* = 131 cases (11 raters [police officers, mental health professionals or doctoral students in forensic psychology, human resource professionals or police analysts ranging from 2-20 years of experience] from the U.S. and Canada were randomly assigned 12 hypothetical homicide cases each) | 10 |
| Miller, 2014 | Domination, control, torture, humiliation | Serial homicide | N/A (commentary/review) | 10 |
| Morgenbesser et al., 2008 | Robbery, elimination, sexual | Triple spree | *n* = 1 offender (case study of a triple spree homicide that occurred in California during a short time frame in 2003)  *n* = 3 victims (robbery as primary motive for first victim, sexual homicide as primary motive for other 2 victims) | 10 |
| Morrall, 2006 | Lust, love, loathing, loot | Mixed | N/A (commentary/review) | 7 |
| Nielsen et al., 2005 | Escalation, intimate, robbery, drug-related | Homicides involving Black or Latino victims or homicides beginning with a physical confrontation | *n* = 1,494 victims (1,033 in Miami across 196 census tracks and 461 in San Diego across 70 census tracks between 1985 and 1995; 334 of the total 1,367 homicides in Miami omitted and 466 of the total 927 homicides in San Diego omitted due to unknown/unconsidered motives) | 10 |
| Nielssen et al., 2007 | Drug, delusions, psychosis | Mixed | *n* = 93 victims  *n* = 88 offenders who experienced acute mental illness | 9 |
| Parker & McKinley, 2018 | Homicide event motive | Mixed | Unknown number of victims and offenders/suspects. Motives were gleaned from existing motives based on data from the U.K.’s *Murder Investigation Manual* (ACPO, 2006)^1^ | 8 |
| Pelletier, 2017 | Drug, gang, dispute, robbery, domestic | Mixed | *n* = 367 cases (based on narratives obtained from the Newark Police Department homicide unit dataset) | 10 |
| Petreca et al., 2023 | Sadism, intense emotions, practical, altered cognition | Asphyxiation / strangulation | *n* ≥ 200 victims (data was obtained from Radford-FGCU Serial Killer Database on homicide cases in the United States and Canada occurring during or after 1970)  *n* = 200 offenders | 9 |
| Pizarro & McGloin, 2006 | Gang-related | Mixed | *n* = 342 victims (205 nongang-related; 137 gang-related; homicides that occurred between January 1999 and July 2004 with data via the Greater Newark Safer Cities Initiative; 69 occurred in 1999, 57 in 2000, 90 in 2001, 65 in 2002, 84 in 2003, 52 in 2004; 75 of the 417 cases were excluded for unknown gang involvement)  *n* = 401 suspects (242 nongang-related; 159 gang-related) | 9 |
| Pizarro et al., 2007 | Domestic, interpersonal dispute, drug-related, gang-related, robbery | Homicide where 3 locations are known (victim address, suspect address, incident address) | *n* = 363 suspects (410 with other motives)  *n* = 363 victims (410 with other motives) | 9 |
| Pizarro, 2008 | Domestic, drug, robbery, interpersonal dispute, other | Mixed | *n* = 659 victims | 9 |
| Pointon & Wright, 2023 | Instrumental-defensive, instrumental-predatory, expressive-affective | Homicide involving Dismemberment | *n* = 71 cases | 8 |
| Raj & Reddy, 2023 | Expressive, instrumental | Mixed | *n* = 1,065,024 victims (data based on homicides occurring in India from 1990-2020) | 10 |
| Razali et al., 2022 | Revenge / jealousy, abuse, distress, neglect, rape/burglary, substance / alcohol, intoxication, poisoning, unknown | Child homicide | *n* = 349 (children ages 18 years and younger)  *n* = 458 offenders | 8 |
| Resnick, 1969 | Altruistic, acutely psychotic, unwanted child, accidental, spouse revenge | Filicide | *n* = 131 cases (88 mothers, 43 fathers) | 9 |
| Rios et al., 2023 | Family-related, sex crime, robbery, criminal activity | Femicide | *n* = 459 victims | 9 |
| Roma et al., 2012 | Romantic jealousy, mercy, altruistic, family / financial / social stressors, retaliation, other, unspecified | Homicide-suicide | *n* = 662 homicide-suicides (resulting in 1776 deaths) | 9 |
| Rosenfeld et al., 1999 | Gang-related | Mixed | *n* = 1,365 cases (reported homicides in St. Louis between 1985 and 1995; 707 gang-affiliated homicide events in St. Louis between 1990 and 1995; 621 excluded because of unknown victim-offender relationship)  *n* = 689 offenders (suspects of 443 nongang youth, 131 gang-motivated, 115 gang-affiliated)  *n* = 707 victims (443 nongang youth, 145 gang-motivated, 119 gang-affiliated) | 9 |
| Schmidt et al., 2005 | Concealment of prior homicide, greed, domestic argument, revenge, family honor, jealousy, sexual motives, other | Multiple (≥1 occurring closely in time/place) | *n* = 26 cases (data on “multiple homicides” collected from autopsy records of the Cologne and Bonn Universities’ Departments of Legal Medicine between 1985 and 2000)  *n* = 31 offenders  *n* = 68 victims | 9 |
| Skrapec, 1997 | Distorted sense of entitlement (seeing self as a victim), empowerment (total control and possession), perverse quest for vitality | Serial | *n* = 5 cases (case study interviews of 5 incarcerated males who are serial murderers found via the Correctional Service of Canada) | 9 |
| Sutton, 2023 | No apparent motive, argument / quarrel, financial gain or settling of accounts / debts, jealousy / envy, frustration / anger / despair, revenge, other, unknown | Femicides | *n* = 1,125 cases of female victims | 9 |
| Tardiff et al., 2002 | Proximal and distal causes, drug-related, other crime, disputes | Mixed | *n* = 722 victims (based on records from 1991–1993 obtained by the Office of the Brooklyn District Attorney) | 9 |
| Tita & Griffiths, 2005 | Gang, felony, drug, argument, familial, domestic | Mixed | *n* = 420 victims (homicides that occurred in Pittsburgh, Pennsylvania between 1997 and 1995)  *n* = 420 offenders | 9 |
| Turvey, 2011 | Power reassurance / competency, personal inadequacy, power assertive / entitled, expression of virility, retaliation or displaced anger, cumulative real or imagined wrongs, sadistic or anger excitation, fantasized sexual pleasure, profit / material gain, material/personal gain | Mixed | N/A (commentary/review) | 9 |
| Varano et al., 2004 | Drug-related | Mixed | *n* = 175 cases (homicides that occurred in Detroit between 1999 and 2002; of which 129 in one of the 13 precincts, 46 as a random sample of citywide cases; of which 88 had no drug presence, 54 had peripheral drug presence, 33 were drug motivated) | 8 |
| Williams, 2023 | Personal gratification, anger, criminal enterprise, financial gain, ideology, power / thrill, psychosis, sexually driven | Serial homicide | N/A (commentary/review) | 9 |
| Yoon et al., 2012 | Affectively impulsive, psychotic, threat / control override, delusional misidentification, infidelity delusion, obey to commanding hallucination, altruistic, confused, religious delusion | Mixed | *n* = 219 homicide offenders with bipolar I disorder (190 had experienced more manic-type symptoms; 29 had experienced more depressive-type symptoms) | 9 |
| Zeoli et al., 2015 | Escalating disputes, revenge, intimate, nonintimate familial, drug, gang, robbery | Mixed | *n* = 560 cases (homicide data from the Newark Police Department between 1997 and 2007; 126 escalation/dispute, 120 drug-motivated, 107 revenge, 75 robbery, 48 intimate partner, 42 nonintimate familial, 42 gang-motivated) | 9 |
| *Note:* CASP Rating = Overall quality assessment rating score (0-10) from the Critical Appraisal Skills Programme Qualitative Checklist. | | | | |
